# Effects of chronic betaine supplementation on performance in professional young soccer players during a competitive season: a double blind, randomized, placebo-controlled trial

**DOI:** 10.1186/s12970-021-00464-y

**Published:** 2021-10-18

**Authors:** Hadi Nobari, Jason M Cholewa, Alfonso Castillo-Rodríguez, Mehdi Kargarfard, Jorge Pérez-Gómez

**Affiliations:** 1 Department of Exercise Physiology, Faculty of Sport Sciences, University of Isfahan, 81746- 7344 Isfahan, Iran; 2 Department of Exercise Physiology, College of Health Sciences, University of Lynchburg, 24501 Lynchburg, VA USA; 3 Department of Physical Education and Sports, University of Granada, 18010 Granada, Spain; 4 HEME Research Group, Faculty of Sport Sciences, University of Extremadura, 10003 Cáceres, Spain; 5 Department of Exercise Physiology, Faculty of Educational Sciences and Psychology, University of Mohaghegh Ardabili, 56199-11367 Ardabil, Iran

**Keywords:** Anaerobic peak power, CMJ, Football, RSA, VO_2max_, Young sports, 1-RM

## Abstract

**Objective:**

Various nutritional strategies are adopted for athletes to maintain and to improve performance during the competition season. Betaine may enhance performance during a competitive season by increasing the testosterone to cortisol ratio and reducing systemic inflammation. The aim of this study was to investigate the effect of betaine supplementation on the bio-motor abilities in young professional soccer players.

**Methods:**

Twenty-nine young professional soccer players (age, 15.5±0.3 years) were matched by position and randomly assigned to one of two groups for 14 weeks: betaine (BG, 2 g/day; *n*=14) or placebo (PG *n*=15). Diet was standardized by a nutritionist, and measures of muscular power (countermovement jump: CMJ), change of direction: modified 5-0-5), acceleration (10 m sprint), sprint performance (30 m sprint time: SpT), muscular strength (leg press and bench press one repetition maximum: 1-RM), repeated sprint ability (running-based anaerobic sprint test: RAST), and aerobic capacity (30-15 intermittent fitness test) were assessed in the pre (P1), mid (P2) and post (P3) season over the course of 5 days. All subjects participated in one soccer match and five training sessions per week.

**Results:**

Significant (*p* < 0.05) group x time interactions were found for maximal oxygen uptake (VO_2max_), anaerobic peak power, and muscular strength favoring BG at P2 and P3 compared to P1. There were meaningful (*p* < 0.05) group x time interactions for CMJ, SpT, and peak power during the RAST that favored the BG.

**Conclusions:**

14-week of betaine supplementation increased predicted 1-RM, VO_2max_, and repeated sprint ability performance in youth professional soccer players. Betaine supplementation seems to be a useful nutritional strategy to improve and to maintain performance during a competitive soccer season.

## Introduction

Soccer is the most popular spectator and participator sport worldwide [1], with over 15 million youth athletes playing annually [2], and 21 % of youth athletes highly specializing in the sport of soccer [3]. Soccer is a high-intensity intermittent sport, requiring athletes to execute a variety of explosive technical and tactical movements repetitively, whereby 75 % of energy production in matches is provided from aerobic metabolism, while anaerobic metabolism comprises the remaining 25 % [4], and up to 2000 kcal may be expended over the course of a match [5]. Standard youth soccer matches consist of 2 halves of 45 min each, separated by a 15-minute break. European and National Leagues’ youth soccer matches cover approximately 8-9 km/h^−1^ per game, of which approximately 500 m are at speeds greater than 19.8 km/h^−1^, with 30-35 sprints, over 120 rapid changes in acceleration/deceleration [6], and generating plasma lactate concentrations over 8 mmol/L [7].

The development of high physical fitness levels early in the soccer season and the ability to preserve those levels over the course of the season are critical for success [8–10]. Professional youth soccer seasons generally involve one match per week and 5 practice/training sessions per week comprised of resistance training, speed and agility training, tactical training, and short sided games [11]. Several studies have reported increases in aerobic and anaerobic fitness over the course of a season in U16 soccer players [12–14], and these improvements in performance appear to be directly related to maturation and the volume imposed by training loads [9]. On the other hand, intense training, competitions, and match related stress result in residual fatigue that may persist throughout the season. Markers of muscle damage (creatine kinase, myoglobin), inflammatory biomarkers (interleukins, C reactive protein, tumor necrosis factor alpha), increases in oxidant bio-markers and reductions in endogenous antioxidants, elevated cortisol, and delayed onset muscle soreness (DOMS) have all been reported to remain altered for greater than 72-hours post-match [15–18]. While linear and repeat-sprint abilities are recovered within 48 h following a soccer match, hamstrings peak torque, rate of force production, and eccentric strength, in addition to countermovement jump (CMJ), all remain depressed for greater than 72-hours post-match [19]. Given the high eccentric involvement of the hamstrings in accelerations, decelerations and changes of direction [20, 21], as well as their involvement in the rapid transition from extension to flexion of the hips and knee during kicking [22], meaningful soccer-specific performance decrements may occur over the course of the season due to residual fatigue. This accumulation of fatigue associated with a professional youth soccer season [23] may partially suppress maturation and training induced performance increases in youth soccer players [24].

Proper nutrition plays a critical role in training, match play, and recovery, and it is necessary for growth and development. In addition to optimizing macro- and micro-nutrient intakes [25], dietary supplements and ergogenic aids may also be employed to support adaptation and manage fatigue. Betaine is a modified amino acid consisting of glycine with three methyl groups ((CH3)3 N+CH2COO−) that is found in shellfish, flour, and some vegetables, such as beetroot, spinach, citrus fruit, alfalfa sprouts, wheat bran, wheat germ and beets [26]. From a mechanistic stand point, betaine may enhance recovery from damaging exercise as it has been reported to increase insulin like growth factor-1 (IGF-1) and IGF-1 receptor expression in C2C12 myoblasts [27] and humans [28], and phosphorylating protein kinase B as part of the Akt/mTOR pathway immediately following an acute bout of resistance exercise [28] to promote protein synthesis. An enhancement of protein synthesis may also occur as betaine is an organic osmolyte (a ‘‘compensatory’’ solute) that stabilizes proteins by countering the denaturing effect of perturbing solutes [29, 30]. With regards to the attenuation of fatigue accumulation, regular monitoring of endocrine hormones, complete blood cell counts and inflammatory cytokines may all be used as indicators of stressors associated with non-functional overreaching status [31]. Two weeks of betaine supplementation was shown to reduce AM basal cortisol levels in healthy young men [28], and we found a significant difference in the testosterone to cortisol ratio, compared to placebo, following 14 weeks of betaine supplementation during a professional youth soccer season [32]. In the same cohort of subjects, 14-week of betaine supplementation decreased the concentration of pro-inflammatory cytokines (i.e. IL-6, IL-1B, or TNF-a) and white blood cell counts associated with a professional youth soccer season [33].

Despite these potential mechanisms, studies examining the ergogenic roles of betaine are limited when compared to other ergogenic aids [34]. Betaine supplementation of 7 to 14 days improve repeat sprint ability and power output during cycle sprinting [35], attenuate power loss following 120 min of cycling at 75 % maximum oxygen uptake (VO_2max_) with a 15-minute sprint at the end [36], and reduce thermal sensations during exercise in the heat with a trend toward greater time to exhaustion [37]. On the other hand, 6 weeks of betaine supplementation did not improve CrossFit specific anaerobic performance or 2 km row time in recreational CrossFit athletes [38], neither 2 weeks of supplementation improved anaerobic Wingate performance in untrained men [39]. Additionally, some [38–41], but not all [42] studies, have shown improvements in muscular strength and power with betaine supplementation.

Given that studies found positive effects of betaine in conjunction with structured training [43], and that betaine attenuated markers of non-functional overreaching [32, 33], betaine may enhance fitness adaptations associated with a soccer season. To our knowledge, the longest betaine training study was 9 weeks, and no studies have examined the interaction between team sport training and betaine supplementation. This study is the final installment of a three-part study investigating the effects of betaine supplementation in youth soccer players [32, 33]. The aim of this study was to investigate the effect of betaine supplementation on the bio-motor abilities in young players over the course of 14-week. We hypothesized that, compared to a placebo supplement, betaine will promote greater improvements in anaerobic and aerobic fitness in professional youth soccer players.

## Materials and methods

### Participants

**Fig. 1:**
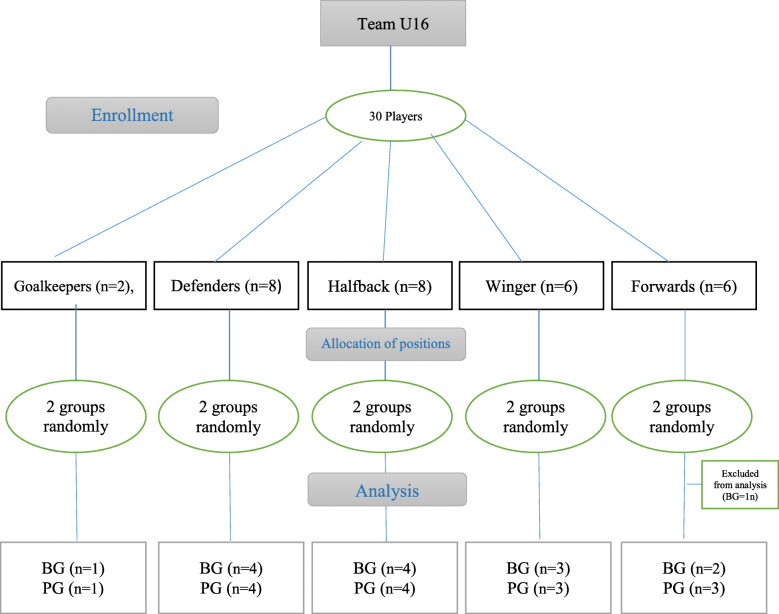
CONSORT chart of study and analysis of participants. BG: Betaine Group; PG: Placebo Group

The participants (*n*=30) were professional young soccer players that competed in the Iranian Youth Premier League for the Foolad Mobarakeh Sepahan Sport Club. Demographics, inclusion and exclusion criteria associated with this sample has been previously published in detail [32, 33]. Briefly, participants had to attend all training sessions and refrain from taking any dietary supplements during the study time or for a year afterwards, abstain from any non-team training, and have no records of sensitivity to dietary supplements in the team medical records. We split the team into five general categories due to the variances in energy systems used in different soccer positions: Goalkeepers (*n*=2), defenders (*n*=8), halfback (*n*=8), winger (*n*=6) and forwards (*n*=6). Subjects were then randomly divided based on position into a supplementation (betaine, *n*=15) or placebo (flour, *n*=15) group [32, 33]. The CONSORT chart can be seen in Fig. 1. A player forward was excluded due to non-compliance with the inclusion criteria from betaine group. Before the trial began, all athletes and their parents were informed of the potential hazards and benefits of participation in the study. To participate in the project, players and their parents signed a consent document. The study was approved by the University of Isfahan’s Ethics Committee before to its launch (IR.UI.REC.1398.102). In this study, researchers based on the Helsinki Declaration (2013) have followed the Human Ethics in Research.

### Experimental approach to the problem

The current study was a semi-experimental, independent group design with pre- (P1), mid- (P2) and post-tests (P3). The participants of the professional club academy were divided to two groups randomly based on their particular positions; betaine group (2 g/day; BG) or placebo group (PG). Betaine anhydrous (TMG, NOW Foods, Bloomingdale, IL) was administered in the capsule form. Players took two capsules per day at two hours prior to and one hour following training with 300 ml water. Players were assessed for their fitness status three times during the season. The P1 was evaluated in the week leading up to the season’s start; the P2, in the seven weeks following the mid-season; and the P3, in the week following the season’s end. For each period of assessment, the players were assessed in five consecutive days. In the first day, assessments of anthropometric, body composition, maturation status, the CMJ, and change of direction (CoD) were performed; On the second day, maximal strength by 1-repetition maximum (1-RM) were measured for the lower and upper body; On the third day, the sprint time (SpT) and acceleration time (AcT) were assessed; while Repeated sprint ability (running-based anaerobic sprint test: RAST), was measured on the fourth day. The aerobic power test was finally performed on the fifth day. During the five-day physical fitness assessments, each player had testing sessions in similar climatic conditions and at the same time [44, 45]. The Newtest Powertimer 300-series testing devise (Newtest Oy, Finland) was used to measure all CMJ, CoD, AcT, SpT and RAST tests, which has demonstrated good reliability for testing both jumping and running variables in young male soccer players [46].

All anthropometric and body composition measurements were obtained between 8 and 11 A.M [47]. All players presented individual wellness questionnaires before the start of each training session, as well as reporting their rating of perceived exertion (RPE) 30 min after each exercise [48–51]. At each stage of evaluated, players recorded their nutrition for three days and gave it to the researchers.

## Procedures

### Team training

Soccer matches for this age group lasted 90 min and were played on an official pitch according to the rules of Iran’s Football Federation. All subjects participated in the following training program: 5 training sessions of 90 min per week, including 10 min of warm-up, 20 min of physical training, 10 min of technical training, 20 min of tactical training, 25 min of training game (including playing in small-sided game), and at the end there was a recovery for 5 min. Strength and power training occurred once per week as part of team training, and consisted of a combination of plyometric, body weight movements and resistance training. Training goals for this age group included goals in small-sided game (development of ball possession, ball transition in speed and rapid organization of zonal defense, retreat and recovery), tactical goals (using defensive and offensive principles quickly), technical goals (focus on passing and controls skills, as well as ball control in small and large spaces), and physical fitness goals (development of aerobic power, linear speed and explosive power) were applied in the exercises of each session.

### Anthropometric and body composition

Detailed procedures for the measurement of anthropometrics and body composition in this sample have been published elsewhere [32, 33]. The researchers used a stadiometer (Seca 213, Germany), a balance scale (Seca 813, UK), and 7 subcutaneous body fat points (Lafayette, USA) and Brozek’s method to determine height, weight, and body composition, respectively [52]. The following formula was used to determine the maturity offset and age at peak height velocity of the players [53] and was previously validated by Mirwald et al. [54]: Maturity offset = −9.236 + 0.0002708 (leg length × sitting height) − 0.001663 (age × leg length) + 0.007216 (age × sitting height) + 0.02292 (Weight by Height ratio).

### Countermovement jump

The CMJ was used to assess lower-body power [55]. A standardized warm-up of 10 to 15 min of jogging was then followed by 5 to 6 sprint specific drills, 1 or 2 CMJs, horizontal bounds and vertical hops, and finally one or two trial jumps for testing familiarization. Participants stood in the center of the contact mat with hands on the hips, they were instructed to rapidly descend until a knee angle of approximately 90 degrees was achieved, and then jump vertically with maximum power. Five minutes of rest was provided between attempts and the best performance was recorded in centimeters [56]. In the CMJ the intra-class correlation (ICC) was 0.96.

### Change of direction

A “modified 5-0-5” [57] was performed five minutes following the CMJ test for CoD. A cone was placed at line “A”, another 5 m away at line “B”, and a third was placed another 5 m away at line “C”. A digital timer connected to photocells placed at hip height were located at line “B”. Subjects stood in a 2-point stance 70 cm behind “A”, sprinted 10 m through line B to line “C”, turned 180 degrees without their hand contacting the ground, and sprinted 10 m back through line B to line “A”. The digital timer began and stopped when subjects passed line “B”. All subjects performed two trials with 3 min of recovery, and the best of the two trials was recorded for the CoD. The ICC for the modified 5-0-5 test was 0.93.

### Muscular strength

To assess 1-RM a predictive test was conducted for the lower and upper body using leg press and bench press, respectively. Each participant completed one practice testing session in order to become familiarized with the test one week prior to testing. During the familiarization session, subjects performed multiple sets with progressively increasing sub-maximal loads to estimate the load used for testing.

Prior to 1-RM testing, subjects performed a general 5-minute low intensity aerobic warm up, then 2 sets of 8 repetitions with 50 % and 75 % of the testing load followed by a 3-minute rest. Subjects were instructed to perform as many repetitions as possible, and the load and repetitions performed were used to estimate the 1-RM. The same load was used in pre- and post-season, unless the subject was able to perform more than 10 repetitions, in which case the load was increased by 10 %. All subjects were given two attempts per exercise with at least 3 min of rest between attempts. For the 45° leg press, feet were positioned at approximately shoulder width apart and subjects were required to descend to 90° knee and 60° hip angle, and fully extend the knee while maintaining contact between the hips and the seat. The bench press was performed according to National Strength and Conditioning Association guidelines [58]. A 1-RM prediction equation was used to estimate the 1-RM based on the load and repetitions recorded [59] as follows: 1-RM= (L). [1.0278- (R × 0.0278)]. Where 1-RM is one maximal repetition, L is the external load in kg, and R is the number of repetitions performed. For leg press and bench press the ICC were 0.91 and 0.93, respectively.

Additionally, volume load was assessed by multiplying the number of repetitions by the load for the bench press and leg press, respectively.

### Acceleration and sprint time

Subjects first performed the same specific, standardized warm up as described in the CMJ procedures. To measure acceleration, a 10 m sprint was performed. Subjects stood in a 2-point stance 70 cm behind the start line where a photocell was placed at hip height. Upon command (Ready, Go!), subjects then sprinted 10 m whereby a second photocell at hip height connected to a digital time recorded the sprint time. The best time of three attempts with 3 min rest between was recorded. Max speed was assessed according to the same protocol, but with a sprint distance of 30 m. For acceleration and sprint tests the ICC were 0.89 and 0.90, respectively.

### Anaerobic test

Prior to the anaerobic power test, subjects first performed the same standardized warm up as described in the CMJ procedures. To measure anaerobic power, a RAST was used. Subjects ran a total of six 35 m sprints separated by 10 s of recovery timed with photocells placed at hip height. The power output of each sprint was calculated according to the previously published formula: Power = (Body mass x Distance^2^) / Time^3^ [60], and the following power variables were also calculated: The highest number recorded called a RAST of peak (RaP); The lowest number obtained called a RAST of minimum power (RaM); The sum of six repetitions divided by six as a RAST of average power (RaA); and RAST of Fatigue Index (RaFi) obtained from “Highest power - lowest power ÷ sum of time 6 sprints” [61]. The ICC of anaerobic power was 0.87, and the ICC for fatigue index was previously reported as 0.70 [60], and previous studies have validated the RAST test [62, 63].

### Aerobic power test

The VO_2max_ was determined using the intermittent Fitness Test 30-15 (30-15_IFT_). Subjects performed a standardized warm up as described in the CMJ procedures, and the 30-15_IFT_ was conducted in groups of four. The procedures as well as baseline results for this sample have been previously published [33]. In brief, the 30-15_IFT_ includes a 40-meter shuttle with 30 s activity and 15 s of recovery at an initial speed of 8 km.h^−1^ and a 0.5 km/h speed increase every 45 s. The test was terminated when subjects could not continue or subjects could not maintain pace for three consecutive shuttles, and the final running speed (VIFT) was recorded. VO_2max_ was estimated with the following formula: VO_2max_ (ml.kg^−1^.min^−1^) = 28.3 – (2.15 × 1) – (0.741 × 16-years) – (0.0357 x body mass) + (0.0586 × 16-years x VIFT) + (1.03 x VIFT). The test-retest of this assessment has been recorded as 0.91 and has been validated in various studies [15, 64–66].

### Dietary monitoring

Dietary monitoring procedures, energy, and macronutrient intake associated with this sample have been previously described in detail [32, 33]. In a nutshell, individuals met with a nutritionist who gave them dietary recommendations for Iranian local foods that delivered 1.55 times their basal metabolic rate in calories. Players used to eat the same items for 72 h before each measure stages and keep track of their intake. To measure compliance, total calorie and macronutrient intake was measured with Nutrition 4 version 3.5.2 software, produced in Iran.

### Statistical analysis

The mean and standard deviation are used to report descriptive statistics. The normality and homogeneity of data variables were checked using the Shapiro-Wilk test and Levene’s test, respectively. A mixed factorial 2 × 2 analysis of covariance (ANCOVA) with repeated measures was used to evaluate all variables. The covariate was considering to the pre-season level variables, the intra subject factor was considering for time (mid- or end-season), and the inter subject factor was considering for group (BG or PG). When a significant time x group interaction was discovered, each group was subjected to a one-way repeated-measures analysis of variance (ANOVA) with the Bonferroni *Post hoc* analysis. If the one-way ANOVA findings for each group were similar, the percent changes for pre-season vs. post-season were calculated and compared using an independent samples t-test. The magnitude of comparisons pre- and post-season for both groups was calculated using Hedge’s g effect size (95 % confidence range). The following are the thresholds: trivial: <0.2, small: ≥ 0.2, moderate: ≥ 0.5, and large: ≥ 0.8. SPSS 22.0 and Graph-Pad Prism 8.0.1 were used for all analyses, and the significance threshold was set at *p* < 0.05.

## Results

**Fig. 2:**
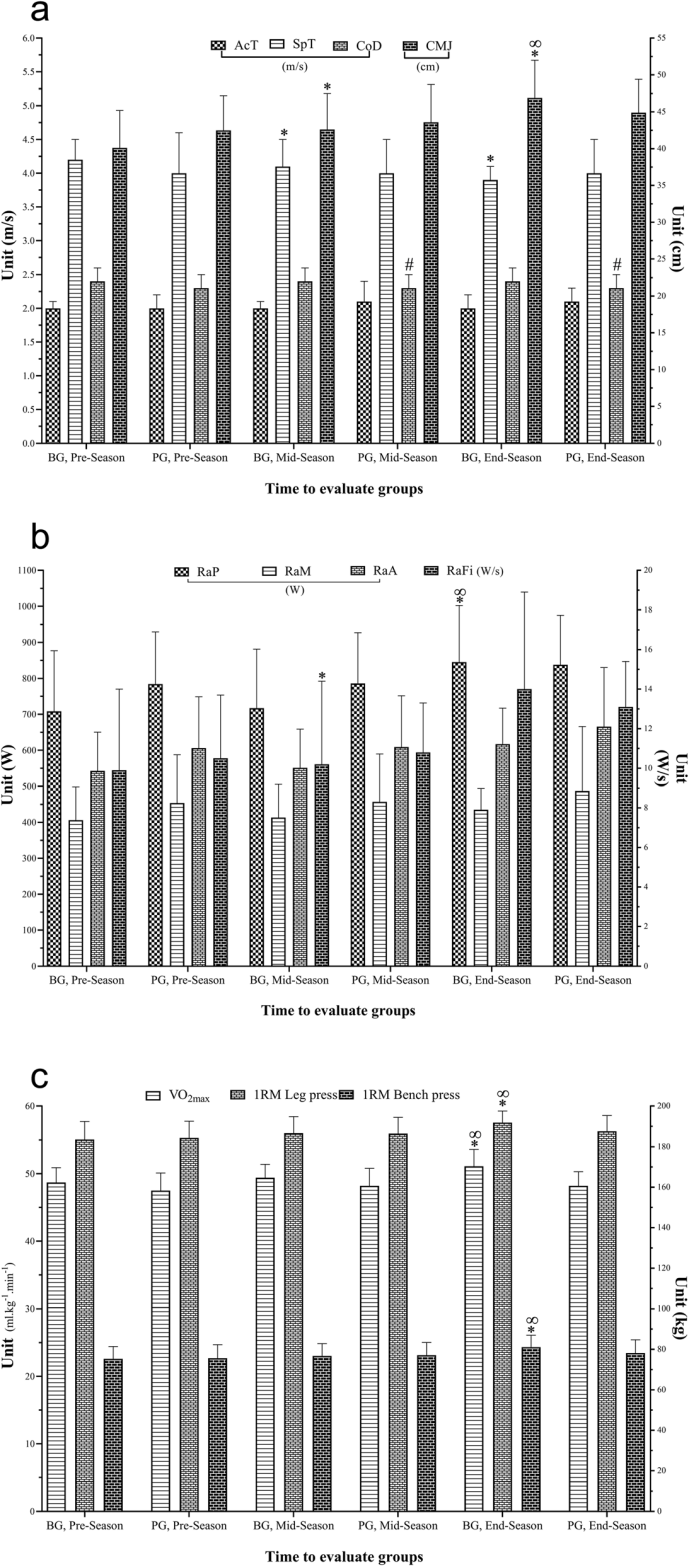
Change in physical fitness assessment for each group and assessment stage. * Represents a statistically significant difference compared to P1 with the superiority of the BG (*p*<0.05); # Represents a statistically significant difference compared to P1 with the superiority of the PG (*p*<0.05); ∞ Represents a statistically significant difference compared to P2 with the superiority of the BG (*p*<0.05); α Represents a statistically significant difference compared to P2 with the superiority of the PG (*p*<0.05); Abbreviation: P1: Pre-season assessments, P2: Mid-season assessments, P3: End-season assessments; BG: Betaine Group; PG: Placebo Group; BG: VO_2max_: Maximal oxygen consumption; CMJ: Counter movement jump; AcT: Acceleration time; SpT: Sprint time; CoD: Change of direction; RaP: RAST of peak power; RaM: RAST of minimum power; RaA: RAST of average power; RaFi: RAST of fatigue index

There were no significant (*p* > 0.05, *F*= 1.87, *η*_*p*_^*2*^ = 0.07) main effects of time for CMJ, but there was a significant group by time interaction (*p* = 0.001, *F*= 14.96, *η*_*p*_^*2*^ = 0.37). *Post hoc* analysis revealed CMJ was significantly (*p* < 0.001) greater at P2 and P3 compared to P1 for BG. For PG, P3 was significantly (*p* < 0.001) greater than P1, and showed a non-significant (*p* = 0.060) trend for P3 compared to P2 (Fig. 2A). Percent changes in CMJ between pre- and post-season were significantly (*p*=0.001) greater in BG than PG (Table 1).

**Table 1:** Changes in physical fitness variables levels between pre- mid- and post-season

Variables	Groups	Pre-Season	Mid-Season	Post-Season	Pre-Post Season		95 % CI Hedge’s g	
		**M±SD**	**M±SD**	**M±SD**	**% Change**	**Hedge’s g**	**Lower**	**Upper**
VO_2max_	BG	48.7±2.2	49.4±2.0^€^	51.1±2.5^*^	4.9	1.0 L	0.17	1.73
(ml.kg^−1^.min^−1^)	PG	47.5±2.6	48.2±2.6^€^	48.2±2.1	1.4	0.3 S	-0.44	0.99
CMJ (cm)	BG	40.1±5.1	42.6±4.9^€^	46.9±5.1^*^	17.1	1.3 L	0.47	2.09
	PG	42.5±4.7	43.6±5.1	44.9±4.5^*^	5.5	0.5 M	-0.25	1.21
AcT (m/s)	BG	1.96±0.14	1.99±0.14	1.99±0.18	1.4	0.2 S	-0.58	0.90
	PG	2.04±0.20	2.12±0.26	2.11±0.23	3.2	0.3 S	-0.44	1.00
SpT (m/s)	BG	4.16±0.34	4.12±0.36^€^	3.92±0.25^*^	-5.8	-0.8 L	-1.54	0.00
	PG	4.01±0.56	4.03±0.54	4.04±0.49	0.8	0.1 T	-0.66	0.77
CoD (m/s)	BG	2.37±0.15	2.40±0.21	2.41±0.22	2.0	0.2 S	-0.51	0.98
	PG	2.29±0.25	2.27±0.24	2.26±0.21	-1.4	-0.1 T	-0.85	0.59
RaP (w)	BG	708.4±168.6	717.1±164.2	845.1±157.0^*#^	19.3	0.8 L	0.03	1.57
	PG	784.2±145.1	785.9±141.2	837.8±137.6^*#^	6.8	0.4 S	-0.36	1.08
RaM (w)	BG	406.1±92.5	413.5±92.0	435.1±59.1	7.1	0.4 S	-0.39	1.10
	PG	453.7±133.6	457.1±132.5	487.1±178.8	7.3	0.2 S	-0.52	0.92
RaA (w)	BG	542.4±107.7	551.0±107.8	616.9±100.0	13.7	0.7 M	-0.08	1.44
	PG	605.9±142.9	608.8±142.8	665.6±164.6	9.8	0.4 S	-0.35	1.09
RaFi (w/s)	BG	9.9±4.1	10.2±4.2	14.0±4.9	41.6	0.9 L	0.09	1.64
	PG	10.5±3.2	10.8±2.5	13.1±2.3	25.1	0.9 L	0.15	1.66
Leg press (kg)	BG	183.6±8.8	186.6±8.2^€^	191.8±5.7^*^	4.5	1.1 L	0.26	1.84
	PG	184.4±8.1	186.4±8.0^€^	187.5±7.8^*^	1.7	0.4 S	-0.35	1.09
Bench press (kg)	BG	75.3±6.1	76.7±6.1^€^	81.1±5.9^*#^	7.7	0.9 L	0.14	1.70
	PG	75.6±6.7	77.1±6.3^€^	78.2±6.5^*#^	3.4	0.4 S	-0.35	1.09
VL on leg press (kg)	BG	1027.7±194.1	1064.4±145.3	1113.2±116.0	11.5	0.5 M	-0.25	1.26
	PG	961.8±193.5	913.6±190.5	940.3±163.5	-0.8	-0.1 T	-0.60	0.83
VL on bench press (kg)	BG	321.0±69.3	326.9±68.5	387.7±63.2	23.1	1 L	0.17	1.74
	PG	395.8±64.8	407.1±70.3	421.5±66.7	7.3	0.4 S	-0.35	1.09

M: Mean; SD: Standard deviation; BG: Betaine Group; PG: Placebo Group; VO_2max_: Maximal oxygen consumption; CMJ: Counter movement jump; AcT: Acceleration time; SpT: Sprint time; CoD: Change of direction; RaP: RAST of peak power; RaM: RAST of minimum power; RaA: RAST of average power; RaFi: RAST of fatigue index; VL: Volume load; P: Pre-Season; P2: Mid- Season; P3: Post- Season; T: Trivial; S: Small; M: Moderate; L: Large

^€^Represents a statistically significant difference compared to P1-P2 (*p*<0.05); ^#^Represents a statistically significant difference compared to P2-P3 (*p*<0.05); ^*^Represents a statistically significant difference compared to P1-P3 (*p*<0.05)

There were no significant group by time interactions for change times in CoD (*p* > 0.05, *F* = 3.48, *η*_*p*_^*2*^ = 0.12), however, there was a significant main effect of time *(p* = 0.009, *F*= 7.86, *η*_*p*_^*2*^ = 0.23) (Fig. 2A).

There were no significant (*p* > 0.05, *F* = 2.99, *η*_*p*_^*2*^ = 0.10) main effects of time for the 1-RM in bench press, but there was a significant group by time interaction (*p* = 0.005, *F* = 64.06, *η*_*p*_^*2*^ = 0.71). *Post hoc* analysis revealed the 1-RM in bench press significantly increased from P1 to P2, P2 to P3, and P1 to P3 in the BG and PG (Fig. 2C). There were significant (*p* = 0.001, *F*= 13.85, *η*_*p*_^*2*^ = 0.35) main effects of time and group by time interactions (*p* < 0.001, *F*= 21.36, *η*_*p*_^*2*^ = 0.45) for changes in 1-RM in leg press. This variable was significantly greater at P3 and P2 compared to P1 in both groups. Percent changes in bench press and leg press 1-RM between pre- and post-season were significantly greater in BG than PG (Table 1).

There were significant (*p* = 0.015, *F* = 6.74, *η*_*p*_^*2*^ = 0.21) main effects of time for the volume load in leg press, but there were no significant group by time interactions (*p* > 0.324, *F* = 1.01, *η*_*p*_^*2*^ = 0.04). There were significant (*p* = 0.007, *F* = 8.57, *η*_*p*_^*2*^ = 0.25) main effects of time, however, there were no group by time interaction (*p* > 0.115, *F*= 2.66, *η*_*p*_^*2*^ = 0.09) for changes in the volume load in bench press.

There were no significant main effects of time for changes in AcT (*p* > 0.05, *F*= 2.753, *η*_*p*_^*2*^ = 0.10) nor group by time interaction (*p* > 0.05, *F*= 0.021, *η*_*p*_^*2*^ = 0.001). There were significant (*p* = 0.003, *F* = 10.35, *η*_*p*_^*2*^ = 0.29) main effects of time and a group by time interaction (*p* < 0001, *F*= 19.43, *η*_*p*_^*2*^ = 0.43) for changes in SpT (Fig. 1A). *Post hoc* analysis revealed SpT was significantly less at P3 versus P1 and P2 only in the BG.

There were significant (*p* = 0.005, *F*= 9.20, *η*_*p*_^*2*^ = 0.26) main effects of time and a group by time interaction (*p* = 0.04, *F*= 4.69, *η*_*p*_^*2*^ = 0.15) for changes in RaP (Fig. 2B). *Post hoc* analysis revealed RaP was significantly greater at P3 compared to P1 and P3 compared to P2 in both groups, however, percent changes between pre- and post-seasons were significantly greater in the BG (*p*=0.035) (Table 1). There were no significant main effects of time for RaM (*p* > 0.05, *F*= 0.22, *η*_*p*_^*2*^ = 0.01) and RaA (*p* > 0.05, *F*= 2.31, *η*_*p*_^*2*^ = 0.08) nor group by time interactions (*p* > 0.05, *F*= 0.09, *η*_*p*_^*2*^ = 0.004) and (*p* > 0.05, *F*= 0.07, *η*_*p*_^*2*^ = 0.003), respectively. There was a significant main effect of time for RaFi *(p* = 0.007, *F*= 8.70, *η*_*p*_^*2*^ = 0.25), but not a group by time interaction RaFi (*p* > 0.05, *F* = 2.95 *η*_*p*_^*2*^ = 0.10).

There were no significant (*p* > 0.05, *F*= 0.06, *η*_*p*_^*2*^ = 0.002) main effects of time for VO_2max_, but there was a significant group by time interaction (*p* = 0.001, *F*= 14.01, *η*_*p*_^*2*^ = 0.35). *Post hoc* analysis revealed VO_2max_, was significantly greater at P2 (*p* = 0.002) and P3 (*p* < 0.001) compared to P1 for BG, but for PG was only significantly (*p* = 0.031) greater at P2 compared to P1 (Fig. 2C).

## Discussion

The study aim was to investigate the effects of betaine supplementation on bio-motor ability in professional youth soccer players throughout a 14-week competitive season. We hypothesized that betaine would lead to greater improvements in performance compared to placebo. The major findings from the study support our hypothesis, with greater improvements in the vertical jump, upper and lower body strength, 30 m sprint, peak power during the RAST test and aerobic performance.

Aerobic endurance, repeated sprint ability, acceleration, lower body muscular strength and power are reported to be the physiological attributes most separating higher-level players from amateurs [67], with muscular strength and power especially being key physiological indicators of performance in professional youth soccer players [68]. The improvements in jumping and running performance occurred irrespective of differences between groups in body composition outcomes [32], suggesting that increases in muscular strength and power production contributed more to these performance improvements than changes in body mass. These results are in line with previous studies that reported significant relationships between lower body strength, CMJ and 20 m sprint in youth soccer players [69]. Given the relationship between lower body strength, power, and heading and tackling success in professional youth soccer players [68], the results of this study suggest betaine supplementation may also enhance on field performance such as jumps, strength, sprints, aerobic and anaerobic performance.

We speculated that betaine supplementation may positively affect fitness adaptations over a competitive soccer season by attenuating the accumulation of fatigue. Although there were no differences in DOMS and Hooper Index items between groups, there were differences in the testosterone to cortisol ratio [32], inflammatory cytokines, white blood cells, and hematological variables that suggest markers of fatigue and recovery were positively affected by betaine supplementation [33]. In regards to muscle function, the hamstrings have been reported to be most affected by a soccer match, requiring greater than 72 h post-match for full recovery [19]. CMJ has also been reported to remain depressed for greater than 72 h post-match [19], and this appears to be due in part to exercise induced muscle damage of the hamstrings [70]. We found the largest differences in effect size between groups in tests with high hamstring involvement, such as the CMJ, 30 m sprint, and peak power during the RAST, which lends further support to our hypothesis that betaine may have affected performance by attenuating fatigue and hastening recovery from muscle damage. Lending support to this hypothesis, Cholewa et al. [71] reported betaine supplementation tended to attenuate decrements in vertical jump following 6 weeks of high-volume resistance training.

Studies that have analyzed the interaction between betaine supplementation and muscular strength and power have reported conflicting results [43]. To our knowledge, only three other studies have investigated the effects of chronic betaine supplementation during training. In two studies, there were no differences increasing upper or lower body 1-RM between groups [43, 72]. In these studies, the training was composed of moderate loads and higher repetitions (~10), and given heavier loads (>85 % 1-RM) seem necessary to maximize strength outcomes [73], the authors suggest that the lack of strength and power specific training may have influenced these outcomes. On the other hand, Tatiana Moro et al. [74] reported increases in squat 3 RM following 6 weeks of CrossFit training. While the training in Tatiana Moro et al. was not standardized, all subjects were required to complete 2-3 CrossFit workouts per week, which typically consist of a muscular strength and power component [75]. In the present study subjects completed one strength and power specific training session per week. Collectively, these results support the hypothesis by Cholewa et al. [72] that strength and power specific training may be necessary to observe an ergogenic effect associated with betaine supplementation.

To our knowledge, this was the second study to look into the benefits of betaine supplementation when used in conjunction with exercise on aerobic performance. In the first study, Tatiana Moro et al. [74] reported no changes in 2000 km row performance (approximately 8.5 min of work) following 6 weeks of CrossFit training and betaine supplementation. It should be noted that the specific mode of aerobic training in this study was not controlled or described in the methods, and that there were no improvements in 2000 km row in either group, which suggests the CrossFit workouts employed may not have been structured or dosed appropriately to increase aerobic capacity. On the other hand, subjects in the present study completed 5 intense training sessions per week that were comprised of running specific to the 30-15_IFT_ test. These contrasting results, in addition to non-significant differences in aerobic capacity when betaine is supplemented without training [76], also lend support to the hypothesis that betaine supplementation must be paired with appropriate exercise training to confer an ergogenic effect. In regards to potential mechanisms, intracellular betaine defends citrate synthase, the first rate limiting enzyme in the Krebs cycle [77], against thermos-denaturation [78]. This likely leads to greater muscle oxygen consumption, as evidenced by the reported reduced muscle tissue oxygen saturation despite increased muscular endurance with betaine supplementation [41], and may partially explain the increase in aerobic performance observed in this study.

A unique strength to this study was the duration and the measurement of performance variables mid-way through the season (following 7 weeks). No studies have investigated the effects of betaine supplementation with respect to fitness and performance for a duration greater than 10 weeks in length, nor have the time course of betaine supplementation been investigated. In the present study, CMJ, 30 m sprint, and aerobic performance were all greater in betaine compared to placebo at week 7, and remained elevated through week 14. On the other hand, improvements in leg press 1 RM and peak power during the RAST in the betaine group did not exceed the PG until week 14. While speculating on mechanisms that may explain these differences is outside the scope of this study, it does highlight the need for more research into the time-course of adaptations associated with betaine supplementation.

There are three main limitations to this study: (i) we were unable to obtain tissue samples to elucidate any bio-molecular mechanisms, such as differences in the activities of muscle protein anabolic and catabolic pathways; (ii) although we monitored training load and indices of well-being, we were not able to measure external workloads by Global Positioning System devices; (iii) a third limitation of this study is the estimation of bio-motor ability assessments over direct measures. Future research should examine the effects of betaine supplementation on exercise induced muscle damage and the bio-molecular mechanisms that may underpin enhanced recovery with betaine supplementation.

## Conclusions

Fourteen weeks of soccer competition and training led to an estimated increase in strength, power and endurance in youth professional soccer players, betaine supplementation accentuated these performance gains. These results imply that betaine supplementation could be a beneficial nutritional strategy to enhance muscular performance, and, together with increases in the testosterone to cortisol ratio reported in this same sample [32], suggest supplementation with betaine may be used as part of a nutritional plan to improve metrics of soccer-specific fitness during a competitive season in youth athletes.

## Data Availability

The datasets used and/or analyzed during the current study are available from the corresponding author on reasonable request.

## Abbreviations

CMJ: counter movement jump
CoD: change of direction
1-RM: 1-repetition maximum strength
SpT: sprint time
AcT: acceleration time
RAST: running-based anaerobic sprint test
30-15_IFT_: intermittent Fitness Test 30-15
VO_2max_: maximum oxygen uptake
RPE: rating of perceived exertion
s-RPE: session rating of perceived exertion
DOMS: delayed onset muscle soreness
RaP: RAST of peak power
RaM: RAST of minimum power
RaP: RAST of average power
RaFi: RAST of Fatigue Index
ICC: intra-class correlation
BG: betaine group
PG: placebo group
P1: pre-season
P2: mid-season
P3: post-season
